# Diagnostic stewardship for human immunodeficiency virus (HIV) testing using computerized physician order entry

**DOI:** 10.1017/ash.2023.188

**Published:** 2023-06-22

**Authors:** Eli P. Wilber, Bhavin B. Adhyaru, Yun F. Wang, Celeste Sellars-Williams, Paulina A. Rebolledo

**Affiliations:** 1 Division of Infectious Diseases, Department of Medicine, Emory University School of Medicine, Atlanta, Georgia; 2 Division of General Internal Medicine, Department of Medicine, Emory University School of Medicine, Atlanta, Georgia; 3 Grady Health System, Atlanta, Georgia; 4 Department of Pathology and Laboratory Medicine, Emory University School of Medicine, Atlanta, Georgia; 5 Grady Health System, Clinical Laboratory, Atlanta, Georgia; 6 Hubert Department of Global Health, Rollins School of Public Health, Emory University, Atlanta, Georgia

## Abstract

Altering the appearance of a computerized physician order entry (CPOE) interface reduces misuse of an HIV diagnostic test by 87%, demonstrating that CPOE design is a key component of diagnostic stewardship. Collaboration between infectious disease providers, clinical laboratorians, and information technology (IT) professionals can result in improved quality and decreased costs.

Diagnostic stewardship is the process of studying and iteratively improving the ordering, performing, and reporting of diagnostic tests to ensure that the right diagnostic test is ordered for the right patient at the right time.^1,2^ This approach can be applied to all diagnostic tests; however, infectious disease tests are at high risk for misuse due to the inherit complexity of the field and the large number of nonspecialists ordering infectious disease diagnostics.^3^ Additionally, the implementation of electronic health records (EHRs) and the associated use of computerized physician order entry (CPOE) have removed barriers to ordering complex and expensive diagnostic tests thus perpetuating diagnostic waste.^4^ This trend has led to calls for EHR optimization and CPOE-embedded interventions that make it “easier to do the right thing.”^1^ We demonstrate the potential for collaborative teams of frontline clinicians and clinical laboratory experts to design and implement these interventions.

The qualitative human immunodeficiency virus (HIV) DNA polymerase chain reaction (PCR) assay is a reference lab developed test mostly used to assist in diagnosing perinatal HIV infection.^5^ It has minimal utility in the diagnosis or management of HIV infection in adult patients but can be confused with the quantitative HIV RNA PCR assay, which is used for the routine management of patients living with HIV. At our institution, HIV DNA PCR is performed as a third-party test at significant cost ($225.60 per test, personal written communication) and, in the preintervention period, adult patients (aged >18 years) comprised 29% of the total test volume (135 of 461 tests). Based on our anecdotal clinical experience, we hypothesized that this high rate of inappropriate ordering was being driven by the appearance of the test in the electronic medical record (Fig. 1), which displayed HIV DNA PCR first due to alphabetical order and not by clinicians deliberately seeking out the incorrect test. We therefore chose to focus on a CPOE-based intervention as opposed to other methods (eg, provider education) with the aim of reducing use of the HIV DNA PCR test in adult patients.

**Figure 1.**
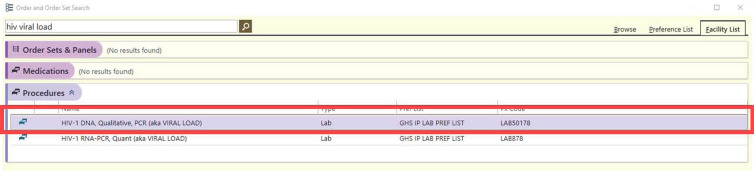
Screenshot of the HIV DNA PCR order in the preintervention state.

## Methods

This study was performed at Grady Health System, a large (>900 bed), urban, academic, safety-net hospital in Atlanta, Georgia. The intervention consisted of modifications to the appearance of the HIV DNA test in the EHR (Epic Systems, Verona, WI). Briefly, the standalone HIV DNA PCR order was removed and only offered through an order set that contained decision-support language indicating that HIV RNA PCR is the appropriate test for adult patients. No “hard stop” was created, and HIV DNA PCR remained available to all patients albeit with additional “clicks.” In the immediate postintervention period, we conducted an audit of HIV DNA PCR orders to identify any patterns of persistent misuse. One provider with a pattern of persistent ordering of HIV DNA PCR in adult patients received targeted education.

We analyzed the impact of our intervention by calculating the average daily number of HIV DNA PCR tests completed on patients aged >18 years using a retrospective pre- and postintervention design. The preintervention period was 365 days and the postintervention period was 180 days. The pre- and postintervention rates were compared by calculating a relative rate ratio. Also, 95% confidence intervals were calculated using the Poisson distribution (https://statpages.info/confint.html). Predicted cost savings were calculated by multiplying the total cost of all adult testing from the preintervention period by the observed relative rate reduction. This project was IRB exempt as a quality improvement initiative.

## Results

**Figure 2.**
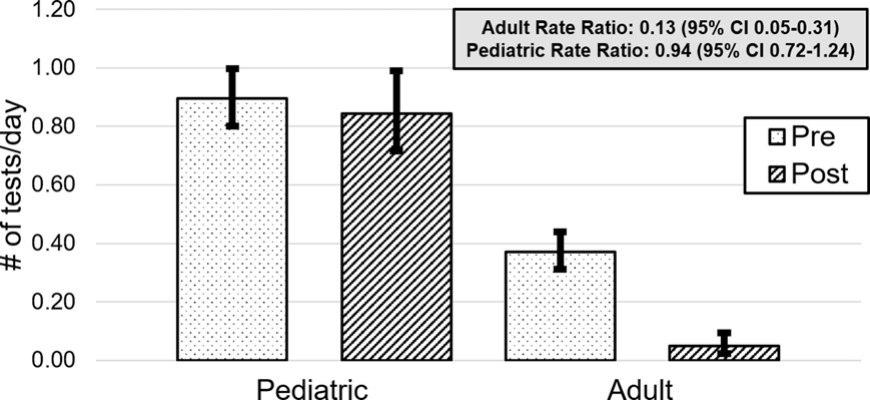
Rates of HIV DNA PCR testing in the pediatric and adult populations in the pre- and postintervention periods, error bars reflect 95% confidence intervals.

In the first 180 days following implementation of the intervention, we observed an 87.5% relative reduction in the rate of adult HIV DNA PCR ordering compared to the preceding 365-day period (0.05 tests per day vs 0.37 tests per day; rate ratio, 0.13; 95% CI, 0.05–0.31) (Fig. 2). There was no significant change in the rate of HIV DNA PCR ordering for pediatric patients. The observed reduction resulted in an ongoing predicted annual savings of $26,350 (95% CI, 21,160–28,870) to our institution.

## Discussion

We implemented a CPOE-based intervention and reduced inappropriate use of the HIV DNA PCR assay in adult patients at our institution by >85%, resulting in >$26,000 a year in predicted variable cost savings. This project required the combined expertise of frontline clinicians, laboratory personnel, and information technologists, and it demonstrates the value of fostering an environment in which these groups can collaborate on quality improvement initiatives. This experience highlights the valuable perspective of frontline clinicians who are well suited both to recognize areas in need of diagnostic stewardship as well as the proximal root causes of diagnostic inefficiencies.

It is natural to analogize diagnostic stewardship to the still relatively young field of antimicrobial stewardship, and indeed, the 2 are complementary. In the last 25 years, antimicrobial stewardship programs (ASPs) have gained widespread endorsement by professional societies and regulatory bodies including formal staffing recommendations from the Centers for Medicare and Medicaid Services.^6,7^ However, despite these advances, there are still substantial challenges in securing adequate funding to support antimicrobial stewardship efforts in both the inpatient, ambulatory, and long-term care settings.^7^ Diagnostic stewardship does not yet have the same regulatory mandate as antimicrobial stewardship but may be at a structural advantage relative to ASPs when it comes to demonstrating the cost savings associated with successful interventions.^8^ A successful diagnostic stewardship intervention, such as the one outlined in this report, will have easily measurable reductions in variable costs directly incurred by the hospital in addition to the potential (and harder to measure) downstream benefits related to fewer misdiagnoses (eg, shortened length of stay, fewer readmissions). In contrast, the benefits of ASPs may be both direct (eg, decreased or less expensive antimicrobial use) as well as more indirect and difficult to attribute (eg, increased patient turnover, fewer nonreimbursable hospital-acquired infections).^8^

Currently, institutional diagnostic stewardship efforts are largely housed within antimicrobial stewardship programs and consequentially focus on issues related to antimicrobial use such as urine-culture practices and *Clostridioides difficile* testing.^1,2^ This trend highlights the prominent role of infectious diseases (ID) physicians in shaping this emerging field and highlights the opportunity for the field of ID represented by further investment in diagnostic stewardship. Cognitive specialties, such as ID, are at a disadvantage in the current fee-for-service model of healthcare delivery in place in the United States, and this relative disadvantage contributes to poor recruitment to the field.^9,10^ Leadership in diagnostic stewardship represents an opportunity for growth for ID physicians by demonstrating their financial value to institutions while at the same time acting as patient advocates. Additionally, the increasingly rapid development of complex and expensive diagnostic tests (eg, multiplex syndromic panels) will increase pressure on payors to demand a more judicious use of diagnostic resources. ID physicians are ideally suited to lead this effort.

This study had several limitations. It reports a single stewardship intervention at a single center. However, the underlying concepts of CPOE structure influencing clinician behavior and the success of a multidisciplinary diagnostic stewardship team are broadly applicable. These positive results should stimulate investment in similar efforts at other institutions and incentivize collaborations between frontline clinicians, laboratory experts, and information technology experts. Additionally, the specific issue of the impact of EMR and CPOE design on clinician behavior, and consequently patient outcomes, deserves further study.
